# Progress and challenges in implementing non-communicable disease policies in Sudan

**DOI:** 10.1186/s12961-023-01079-2

**Published:** 2023-12-06

**Authors:** Yasir Ahmed Mohammed Elhadi

**Affiliations:** 1 Department of Public Health, Sudanese Medical Research Association, Khartoum, Sudan; 2 Division of Healthcare Policy and Finance, Global Health Focus, Khartoum, 1111 Sudan

**Keywords:** Non-communicable diseases, Health policy, Health system reform, Policy analysis, Sudan

## Abstract

Non-communicable diseases (NCD) pose a substantial global public health challenge, representing the leading cause of morbidity and mortality worldwide. This study investigates the progress and challenges in implementing NCD policies in Sudan. Document analysis following the ready your materials, extract data, analyse data and distil your findings (READ) approach, was utilized to review published literature and reports. Data from the NCD Progress Monitor showed that the percentage of NCD-related mortality had increased from 32% in 2015 to 54% in 2022. Sudan’s progress in implementing NCD policies has been slow and challenging; eight of the 19 NCD target indicators had never been fully achieved, and only five targets were fully achieved in the year 2022. However, these figures may be underestimated due to the lack of robust NCD information systems. Like many countries, Sudan faces challenges in implementing NCD policies, particularly those targeting healthy diets, medications and data management systems. This may be linked to the prolonged history of conflict, shortage of trained health personnel, limited resources and lack of robust NCD surveillance systems in the country. The ongoing devastating war and destruction of the healthcare system infrastructure in Sudan further intensified these challenges. Prioritizing NCD policies and programmes during the anticipated post-conflict health system reforms is crucial for enhancing NCD prevention and outcomes in Sudan.

## Introduction

Non-communicable diseases (NCD) are the leading cause of death worldwide, accounting for approximately 71% of global deaths. Cardiovascular diseases account for 17.9 million deaths, followed by cancers, respiratory diseases and diabetes. NCD have become a major public health challenge for both low- and middle-income countries (LMICs) and high-income countries [1].

LMICs are experiencing a double burden of disease, with a high prevalence of infectious diseases such as human immunodeficiency virus/acquired immune deficiency syndrome (HIV/AIDS), tuberculosis and malaria, as well as an increasing burden of NCD. This shift is due to changes in lifestyle, urbanization and the adoption of unhealthy diets and behaviours. In LMICs, NCD are responsible for 80% of premature deaths, and the economic burden of NCD is expected to exceed US$ 7 trillion by 2025 [2, 3].

In May 2015, the World Health Organization (WHO) released a technical note detailing its plan for reporting to the United Nations (UN) General Assembly in 2017 regarding the advancements made in fulfilling the national commitments outlined in the 2011 UN Political Declaration and the 2014 UN Outcome Document on NCD. [4] This note was later updated in September 2017 to align with the revised set of WHO-recommended strategies and interventions, known as ‘best buys’, for preventing and managing non-communicable diseases. These ‘best buys’ were officially endorsed by the World Health Assembly in May 2017 [5].

The NCD policies are focussed on behavioural and metabolic risk factors for NCD, including tobacco control, reducing harmful alcohol consumption, promoting physical activity and improving access to healthy diets as well as strengthening health systems to improve early detection and management of NCD [6, 7]. Evaluation of policy implementation and effectiveness is critical to address the NCD epidemic in LMICs. Nevertheless, there is still a lack of focussed health research and development efforts in many LMICs concerning NCDs despite repeated calls [8]. The purpose of this article is to highlight the progress and challenges in implementing NCD policies in Sudan.

## Methods

The document analysis methodology was adopted to assess the implementation of NCD policies in Sudan. Document analysis is commonly used in health policy analysis studies [9], and for this study, the ready your materials, extract data, analyse data and distil your findings (READ) approach was followed [10].

The WHO Progress Monitor reports published in 2015 [11], 2017 [12], 2020 [13], and 2022 [14] and published Ministry of Health reports in Sudan formed the basis for the analysis. The country-level data were extracted and compared to highlight the progress in implementing NCD policies in Sudan. This method has been previously utilized to assess the progress on NCD policy implementation at the global [15] and national levels [16].

In addition, a thorough review of published literature and reports across the PubMed, Google Scholar and Google databases was conducted to identify and contextualize the challenges faced in implementing NCD policies and programmes in Sudan. Specific information related to the implementation of NCD policies, progress indicators and challenges were extracted, and a detailed analysis of the gathered information was conducted. The analysis involved categorizing the data, identifying patterns and trends, and critically evaluating the content to distil a coherent insight into the progress and challenges of NCD policy implementation in Sudan.

## Progress in implementing NCD policies in Sudan

**Fig. 1:**
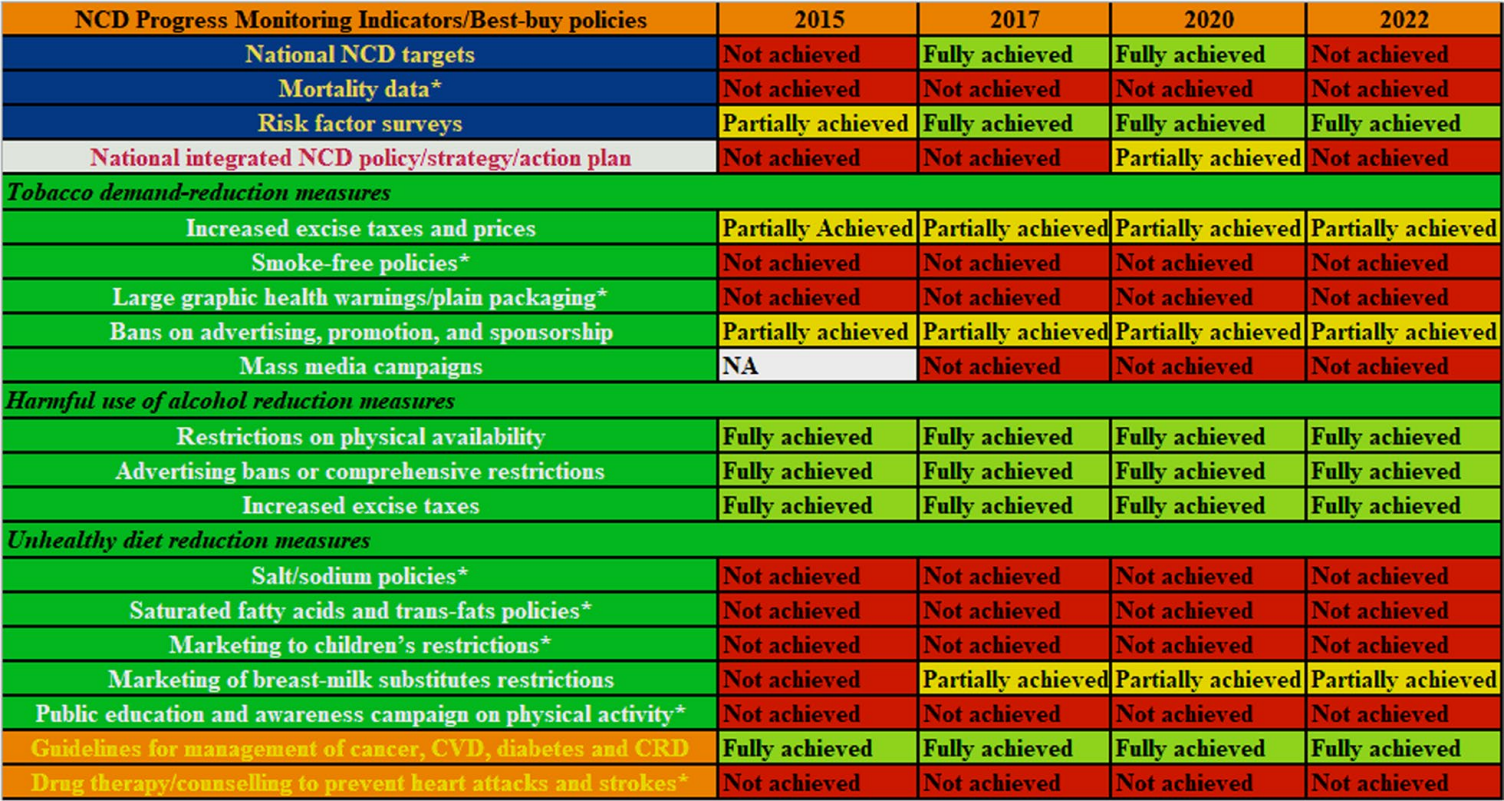
Progress in implementing NCD policies in Sudan 2015–2022. *Never fully achieved; NA, not available

Sudan’s progress in implementing NCD policies has been slow and challenging. Figure 1 shows a joint analysis of NCD progress monitoring indicators in Sudan published in the period between 2015 and 2022. A total of 8 of the 19 WHO NCD indicators had never been fully achieved and only 5 indicators were fully achieved in 2022. This progress is relatively slow compared with other countries in the African region [17, 18]. Sudan faces challenges in implementing NCD policies particularly those targeting healthy diets, medicine and information and monitoring systems (Fig. 1).

### National NCD targets, mortality data and risk factors surveys

**Fig. 2:**
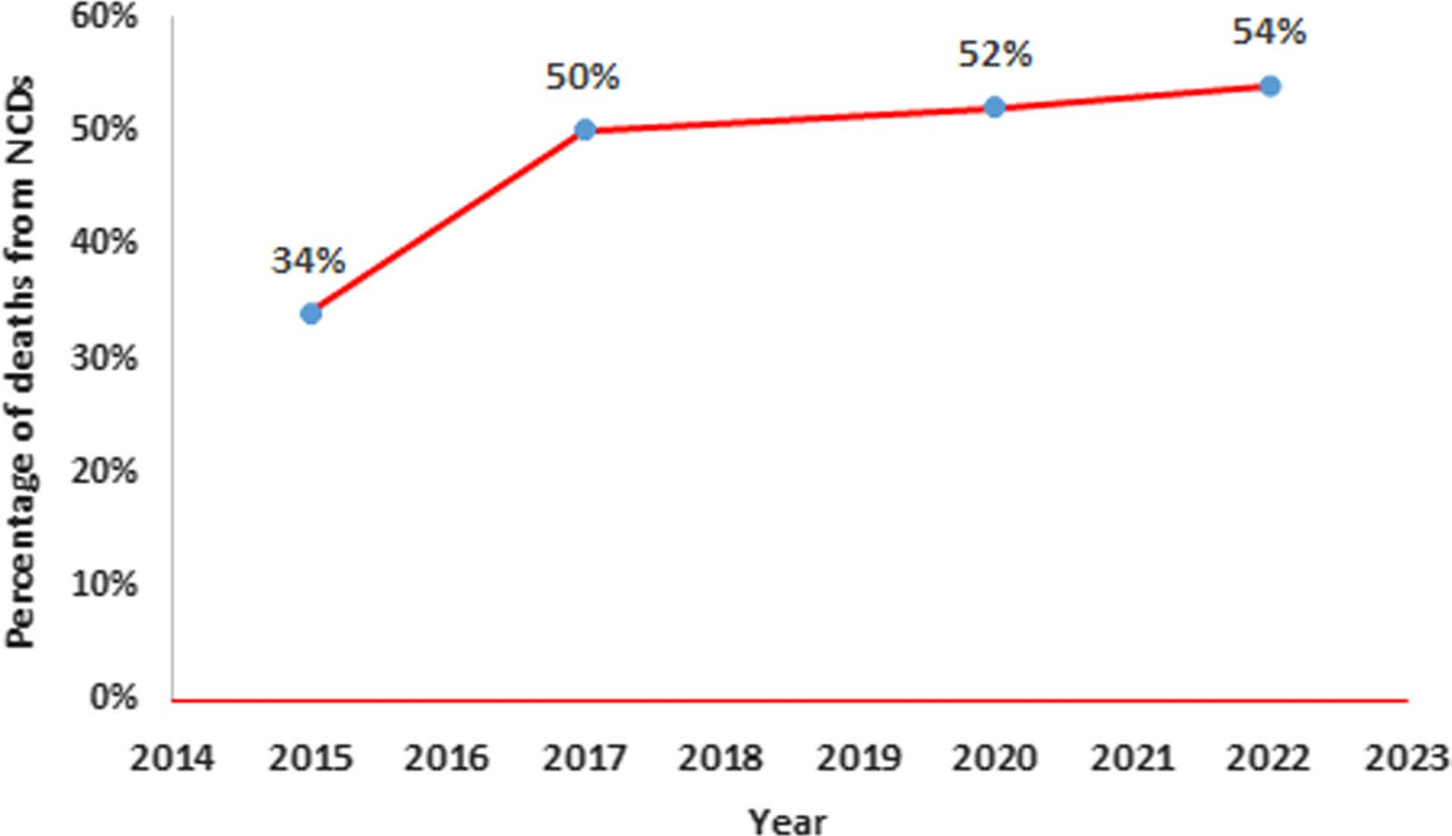
Non-communicable diseases related mortality in Sudan 2015–2022

Drawing on the National Multi-Sectoral Action Plan for the Prevention and Control of NCD (2015–2020) in Sudan, the national target for premature mortality from NCD was to achieve a 25% reduction in overall mortality from cardiovascular diseases, cancer, diabetes or chronic respiratory diseases by 2020 (baseline 2014) [19]. However, data from the Progress Monitor reports showed that the percentage of NCD-related mortality had increased from 32% in 2015 to 52% in 2020, and it was at 54% in 2022 (Fig. 2). In addition, the indicator of NCD targets was not achieved in 2022, although it was fully achieved in 2017 and 2020. Furthermore, the indicator of setting a national integrated NCD policy, strategy and action plan was partially achieved in 2020 and not achieved in 2022 (Fig. 1). Nonetheless, it is important to note that progress towards implementing NCD policies can be slow and incremental, and it may take time to see the full impact of these policies on NCD rates and outcomes. The WHO STEPwise approach to NCD risk factor surveillance in Sudan represents a concerted effort to systematically assess the prevalence of NCD risk factors through standardized data collection methods and collaboration with Sudan’s Ministry of Health and stakeholders, it has yielded a valuable repository of information, informed evidence-based policies and raising public awareness about NCD prevention [20].

### Harmful use of alcohol and tobacco demand reduction measures

Policies concerning alcohol are strictly implemented in Sudan, aided by the Islamic community in the country, in contrast to many other countries where policies for alcohol reduction were the most likely to have been dropped [15]. Sudan ratified the WHO Framework Convention on Tobacco Control, which is an international treaty aimed at reducing the demand for, and supply of, tobacco products [21]. Although some policies were partially sustained, target indicators concerning tobacco demand reduction had never been fully archived during 2015–2022 (Fig. 1). Moreover, recent systematic review showed a high utilization of tobacco products in Sudan with prevalence ranging from 1–25% and 10–47.5% among adolescents and adults, respectively [22], indicating the need for urgent interventions.

### Unhealthy diet reduction measures

According to the Progress Monitor reports, Sudan had not fully achieved the unhealthy diet reduction measures targets from 2015 to 2022. The targets for sodium intake, saturated fatty acids, marketing for children’s restrictions, and public awareness campaigns on physical activity had never been achieved (Fig. 1). The Sudanese dietary habits play a critical role in shaping public health outcomes, as traditional food consists of unbalanced diet with high levels of carbohydrates and sugars while lacking in essential micronutrients [23]. One study reported that obesity and unhealthy dietary habits among university students in Sudan are a growing concern in the country [24]. In addition, more recent research showed that 22.2% of medical students were overweight and 44.9% with a low level of physical activity [25].

## Challenges

The current state of NCD policy implementation in Sudan is challenging. Despite the high burden of NCD in the country, several factors hindered the effective implementation of NCD policies:

### Limited financial and human resources

Sudan has limited financial and human resources for healthcare and disease prevention, which could make it difficult to implement comprehensive NCD policies and programmes [26]. The overall health budget in Sudan is low [27], and NCD prevention and control programmes received less priority in terms of resource allocation [28]. Additionally, the healthcare system in Sudan has suffered a severe shortage of trained health personnel, particularly in rural areas [29]. This shortage has further hindered the effective implementation of NCD policies and programmes including the provision of necessary care and treatment for NCD [30].

### Inadequate health supplies and information systems

Surveillance systems are essential for target setting, monitoring, planning, raising awareness, emphasizing political commitments and accurately estimating the burden of NCD and the effectiveness of interventions. However, surveillance and monitoring systems for NCD are weak in Sudan. A recent operational assessment of implementing the NCD kit in Sudan’s humanitarian environment revealed that no electronic Health Information System was in use, and no NCD-specific registers or tracing systems were employed. In addition, patient-held record-keeping practices and the unavailability of file retrieval technologies further hampered the evaluation of NCD data [30]. Previous research has shown that inadequate NCD surveillance systems undermine health policy and planning particularly in fragile and conflict settings [16, 31, 32]. Sudan is facing many other challenges to the healthcare system, including limited access to healthcare facilities and health supplies which might hinder the implementation of NCD policies and programmes [33, 34].

### Poor health literacy

Effective NCD policies rely on people’s understanding of the importance of healthy behaviours, regular screening and adherence to prescribed treatments and health recommendations. However, there are low levels of health awareness in Sudan. For example many individuals do not recognize the risk factors associated with NCD [35], which may be due to delayed diagnosis, lack of preventive behaviour and inadequate management of NCD. This lack of awareness also affects healthcare-seeking behaviours resulting in late-stage diagnoses and complications [36]. Also, poor health literacy affects the ability to comprehend and adhere to health recommendations and lifestyle modifications. Studies have reported concerning low levels of adherence to diabetes [37], cardiovascular diseases [38] and hypertension medications and guidelines in Sudan [39–41]. Without adequate health literacy, the essential aspects of NCD prevention and management are compromised.

### Lack of political commitment and coordinated action

The political will to address NCD is lacking in some LMICs due to competing political priorities, a lack of awareness of the impact of NCD, and vested interests [42]. The national political agenda in Sudan does not prioritize health system development including NCD prevention and treatment [43]. This is shared with other African countries where there is limited support and lack of prioritization of NCD prevention and control programmes [16, 18, 44].

### The impact of conflict

The prolonged history of conflict and the recent devastating war have posed formidable challenges to the NCD control programmes and policies in Sudan. The destruction of health infrastructure has resulted in limited access to healthcare services, including preventive, diagnostics and treatment services [45]. Moreover, the displacement of populations, disruption of healthcare delivery and loss of skilled healthcare professionals have compounded the challenges [46]. The destruction of health facilities not only impedes the immediate medical response but also hampers the long-term efforts to tackle NCDs as all pivotal aspects of NCD management become severely compromised [47].

## Limitations

This study encountered several limitations. Qualitative insights from stakeholders were not included due to the ongoing war, hindering a deeper understanding of the challenges faced. Additionally, access to the latest unpublished documents from the Federal Ministry of Health was restricted, limiting the study’s scope to only published documents and reports. Despite these limitations, the findings provide valuable insights into Sudan’s NCD policy landscape, emphasizing the urgent need for comprehensive reforms and international support.

## Conclusion

Sudan’s progress in implementing NCD policies has been slow and challenging, marked by a concerning rise in NCD-related mortality rates. Challenges such as limited financial resources, inadequate healthcare infrastructure and political instability have impeded progress on implanting NCD policies and programmes. Urgent actions are needed, including strengthening healthcare infrastructure, enhancing health literacy and instigating political commitment to prioritize NCD prevention and control. These efforts should be informed by comprehensive, updated national strategies that reflect global interventions and involve multi-sectoral collaboration.

## Data Availability

The datasets used and/or analysed during the current study are available from the corresponding author upon reasonable request.
